# An evaluation of sexual function and health-related quality of life following laparoscopic surgery in individuals living with endometriosis

**DOI:** 10.1093/humrep/deae063

**Published:** 2024-04-01

**Authors:** Leesa Van Niekerk, Shaunagh Pugh, Antonina Mikocka-Walus, Cecilia Ng, Rebecca O’Hara, Mike Armour, Mathew Leonardi, Subhadra Evans

**Affiliations:** School of Psychological Sciences, College of Health & Medicine, University of Tasmania, Hobart, TAS, Australia; National Endometriosis Clinical and Scientific Trials (NECST) Network, UNSW, Sydney, Australia; School of Psychological Sciences, College of Health & Medicine, University of Tasmania, Hobart, TAS, Australia; National Endometriosis Clinical and Scientific Trials (NECST) Network, UNSW, Sydney, Australia; School of Psychology, Faculty of Health, Deakin University, Geelong, VIC, Australia; National Endometriosis Clinical and Scientific Trials (NECST) Network, UNSW, Sydney, Australia; Division of Obstetrics and Gynaecology, School of Clinical Medicine, Medicine and Health, University of New South Wales, Sydney, NSW, Australia; Robinson Research Institute, Adelaide Medical School, University of Adelaide, North Adelaide, SA, Australia; National Endometriosis Clinical and Scientific Trials (NECST) Network, UNSW, Sydney, Australia; NICM Health Research Institute, Western Sydney University, Penrith, NSW, Australia; Robinson Research Institute, Adelaide Medical School, University of Adelaide, North Adelaide, SA, Australia; Department of Obstetrics and Gynecology, McMaster University, Hamilton, ON, Canada; School of Psychology, Faculty of Health, Deakin University, Geelong, VIC, Australia

**Keywords:** endometriosis, health-related quality of life, sexual function, symptom burden, pain, dyspareunia

## Abstract

**STUDY QUESTION:**

What is the relationship between sexual function, health-related quality of life (HRQoL), and laparoscopic surgery in individuals living with endometriosis?

**SUMMARY ANSWER:**

A higher number of laparoscopic surgeries is significantly associated with poorer HRQoL and greater levels of sexual dysfunction in individuals with endometriosis.

**WHAT IS KNOWN ALREADY:**

Prior research indicates that endometriosis is associated with lowered HRQoL and sexual function and that these outcomes are influenced by endometriosis-related symptom profiles, medical, and surgical management. A limited number of studies have examined changes in sexual function in individuals with endometriosis following laparoscopic surgery or following repeated surgeries.

**STUDY DESIGN, SIZE, DURATION:**

A cross-sectional community-based online survey was used to examine the relationships between sexual function, HRQoL, and laparoscopic surgery (n* *=* *210).

**PARTICIPANTS/MATERIALS, SETTING, METHODS:**

Individuals with a self-reported diagnosis of endometriosis were recruited via online advertising through social media and gynaecology clinics. Endometriosis-specific data (e.g. diagnostic delay, symptom experience) was collected in addition to engagement with laparoscopic surgery, level of HRQoL (EuroQol-5 Dimension: EQ-5D-5L), and sexual function (Female Sexual Function Index: FSFI). Bivariate correlational analyses and hierarchical multiple regression were used to determine the associations between the variables of interest.

**MAIN RESULTS AND THE ROLE OF CHANCE:**

Individuals with endometriosis have substantially poorer HRQoL in comparison to Australian normative samples, with greater levels of endometriosis-related symptom burden, distress, and pain significantly associated with lower levels of HRQoL. The mean FSFI score was suggestive of clinically significant female sexual dysfunction, with the lowest level of function noted in the domain of sexual pain and the highest level of function noted in the sexual satisfaction domain. A greater number of laparoscopic surgeries was significantly associated with poorer overall HRQoL and greater levels of sexual dysfunction.

**LIMITATIONS, REASONS FOR CAUTION:**

The cross-sectional nature of the data precludes direct findings of causality and further longitudinal research is recommended. The information pertaining to engagement in laparoscopic surgery was self-report in nature and was not medically verified.

**WIDER IMPLICATIONS OF THE FINDINGS:**

The study’s findings highlight the pervasive impact of endometriosis on all domains of living, emphasizing the need to extend treatment planning beyond that of physical pain management alone. Early referral for assessment and management of sexual wellbeing is recommended prior to, and post-surgical intervention, with a focus on maintaining post-surgical changes, potentially reducing the need for multiple surgeries.

**STUDY FUNDING, COMPETING INTEREST(S):**

The study was not associated with research funding. Author CN reports grant funding from the Australian Government and Medical Research Future Fund (MRFF) and was a previous employee of CSL Vifor (formerly Vifor Pharma Pty Ltd).

**TRIAL REGISTRATION NUMBER:**

N/A.

## Introduction

Endometriosis is a persistent systemic inflammatory condition that impacts approximately one in nine people who were presumed female at birth (Rowlands *et al.*, 2021). Treatment is generally aimed at preventing disease progression and symptom management, with multimodal approaches considered the most effective, which may include hormonal and laparoscopic surgical management (Abbott *et al.*, 2003; Della Corte *et al.*, 2020; Laguerre *et al.*, 2022). Laparoscopic surgery is a minimally invasive surgical technique (ablation or excision) used to remove endometriotic tissue from the internal organs and aims to decrease symptom severity in individuals with endometriosis (Mehedintu *et al.*, 2014). While the difference in outcomes between ablation and excision laparoscopic surgery are still unclear in the literature (Bafort *et al.*, 2020), the goal of surgery is to completely remove endometriotic lesions, for which excision is generally the preferred technique, with some research indicating increased efficacy in managing symptoms such as dyspareunia (Healey *et al.*, 2015; Riley *et al.*, 2019).

The ability to engage with and maintain fulfilling interpersonal relationships is strongly related to psychological wellbeing and is a key aspect of health-related quality of life (HRQoL). Research suggests that sexual satisfaction, orgasmic function, and arousal are all symptoms of endometriosis that are commonly overlooked within clinical settings, especially when considering the heterogeneity of endometriosis symptoms (Fairbanks *et al.*, 2017; Vannuccini *et al.*, 2023). However, the link between poorer sexual function and HRQoL suggests that accounting for sexual satisfaction and its contributors should be a prominent treatment consideration in addressing lowered HRQoL (Dogan *et al.*, 2013; Franck *et al.*, 2018). Overall, the impact of lowered sexual function is strongly associated with multiple domains of HRQoL (e.g. physical, psychological, social, relational), suggesting that sexual function and HRQoL are intrinsically linked for most individuals with endometriosis (van Poll *et al.*, 2020).

Few studies have investigated the improvement in sexual function for individuals with endometriosis following laparoscopic surgery, particularly across time. Similarly, limited research is available regarding the efficacy of repeated surgeries for improving sexual function and HRQoL impacts (D’Alterio *et al.*, 2021). A study by Barbara *et al.* (2017) found that there was an improvement in sexual function following laparoscopic surgery, but that this improvement was likely to be more pronounced if a holistic approach to treatment was adopted, given that physical pain alone is not the sole contributor to decreased sexual satisfaction and function. This is supported by a systematic review conducted by Franck *et al.* (2018), which indicated that sexual function is improved following laparoscopic surgery. However, it was noted within the review that there was substantial heterogeneity within the research in terms of the questionnaires used, the stage of endometriosis, and follow-up duration (Franck *et al.*, 2018). A recent prospective study (n = 193) conducted by Martínez-Zamora *et al.* (2021) found that there was a significant improvement in sexual function 6-month post-laparoscopic procedure, and that this improvement was still present but less significant at 36 months (Martínez-Zamora *et al.*, 2021).

Although it is expected that laparoscopic surgery will lead to an initial reduction in endometriosis-related pain and improved HRQoL and sexual functioning in individuals diagnosed with endometriosis, research investigating surgical outcomes has predominantly focused on changes in sexual pain with limited focus on other aspects of sexual function such as desire and enjoyment (Barbara *et al.*, 2017). As alterations to sexual function are treatment specific, understanding the role that laparoscopic surgery plays in addressing the individual components of sexuality (e.g. desire, arousal, satisfaction) may assist medical practitioners in treatment and referral planning (e.g. engagement in sexual therapy assessment and management pre and/or post-surgery). Furthermore, understanding whether certain endometriosis-related symptom profiles are associated with greater sexual function impairments and lowered HRQoL may also assist in effective treatment planning and management where surgical management may not be readily accessible or is contraindicated.

Therefore, the current study aims to investigate sexual function and HRQoL in individuals with symptomatic endometriosis and the influence of laparoscopic surgery on these outcomes. Based on the existing literature investigating the effects of laparoscopic surgery and symptom improvement (Laguerre *et al.*, 2022), it is hypothesized that sexual function and HRQoL will be significantly negatively associated with the length of time following laparoscopic surgery. To extend the understanding of the relationship between repeated laparoscopic surgeries beyond that of fertility management (Rizk *et al.*, 2015), the current study will also examine the relationship between number of surgeries, HRQoL, and sexual function. Based on the evidence that poorer fertility outcomes may be associated with repeated surgical procedures (Rizk *et al.*, 2015), it is hypothesized that a higher number of laparoscopic procedures will be significantly negatively associated with HRQoL and sexual function. The study will also undertake exploratory analyses to explore whether specific endometriosis-related symptoms, laparoscopic surgery, and HRQoL are significant correlates of sexual function in individuals with endometriosis.

## Materials and methods

### Study population

The study population consisted of 210 individuals, presumed female at birth, with symptomatic endometriosis and an average age of 31.7 years (SD* *=* *7.87, range =* *18**–**50 years). Most of the sample identified as female (n* *=* *203, 96.7%), with seven participants (3.3%) self-identifying as non-binary/gender fluid. The study inclusion criteria were: (i) a self-reported diagnosis of endometriosis, (ii) being aged 18 years or older, and (iii) experiencing at least one endometriosis-related symptom in the previous 4-week period. Participants needed to satisfy all three inclusion criteria to be eligible to participate. Participants were recruited via advertisements at gynaecological health services, targeted social media (e.g. Endometriosis Australia, EndoActive, EndoZone), and snowball sampling. Survey data was collected via the RedCap survey platform, hosted by the University of Tasmania (Harris *et al.*, 2019). Participants were directed to a Participant Information Sheet, with submission of the completed survey constituting informed consent.

### Measures

#### Demographic and endometriosis-related information

Demographic information (e.g. country of residence, education, employment) and endometriosis-specific information (e.g. symptom duration, diagnostic delay, disease stage/grading) were collected at the start of the survey. Participants also indicated the presence/absence of 17 endometriosis-related symptoms in the four weeks prior to survey completion (e.g. dysmenorrhea, dyspareunia, voiding pain, fertility concerns, nausea). The 17 endometriosis-related symptoms were based on a review if the literature of frequently reported/measured symptoms and have been used previously with endometriosis samples (Van Niekerk *et al.*, 2022a,b). Information regarding the number of laparoscopic procedures, date of most recent procedure, and hormonal therapy was also gathered.

#### Subjective endometriosis pain rating

The Visual Analogue Scale (VAS) pain rating was used as a measure of the subjective experience of pain. The VAS consists of a straight horizontal line, where the endpoints define extreme limits of pain, with 0 = No Pain and 10 = Worst Pain. Participants are asked to use a sliding scale to indicate the severity of endometriosis-related pain experienced: (i) over the past 7 days, and (ii) at the time of survey completion. The VAS is a reliable and valid pain measure in endometriosis populations (Bourdel *et al.*, 2019).

#### Health-related quality of life

The EuroQol-5 Dimension (EQ-5D-5L) was used to measure mobility, self-care, usual activities, pain/discomfort, and anxiety/depression (Herdman *et al.*, 2011). Each dimension has five levels: no problems (Level 1), slight problems, moderate problems, severe problems, and extreme problems (Level 5). This creates a possible range of 3125 health states when a rating from each dimension is combined, with 11 111 reflecting optimal health and 55 555 reflecting worst health. The EQ-5D-5L health state can be converted to an index ‘utility’ score based on population-based scoring algorithms. As most of the sample resided in Australia, an Australian scoring algorithm and value set were utilized to provide population-based scores (Norman *et al.*, 2023). Based on this algorithm, values below 0 are considered to represent health states worse than death (Devlin *et al.*, 2018). The EQ-5D-5L also includes a visual rating scale, where the respondent is asked to rate how good or bad their health is at that moment in time, with 0 = Worst Health Imaginable and 100 = Best Health Imaginable. A recently conducted systematic review found the EQ-5D-5L to be a valid and reliable measure of HRQoL across a range of populations, conditions, and settings (Feng *et al.*, 2021).

#### Sexual function

The Female Sexual Function Index (FSFI) is a 19-item self-report measure that provides an assessment of six sexual function domains: desire, arousal, lubrication, orgasm, global satisfaction, and pain (Rosen *et al.*, 2000). Respondents are asked to rate their responses on a scale from 0 (No Sexual Activity) to 5 (Almost Always/Very High), excluding questions 15 and 16 which are rated on a scale from 1 (Very Dissatisfied) to 5 (Very Satisfied), with higher scores reflecting greater levels of sexual functioning in the individual domain. Participants are given a total FSFI score, ranging from 2 (dissatisfactory sexual function) to 36 (satisfactory sexual function). The FSFI was found to be a reliable and valid measure of sexual function across the lifespan and has been utilized in research relating to medical and psychological disorders and their impacts on sexual function (Bartula & Sherman, 2015).

### Design and data analysis

A cross-sectional design was employed to explore the associations between endometriosis-related factors (e.g. symptom duration, average endometriosis-related pain level), sexual function, and HRQoL. Only fully completed and submitted survey responses were included in the data analyses as per the restrictions of the study’s research ethics approval (283 started surveys, 240 completed surveys: 84.81% completion rate). Preliminary data review revealed some statistically significant differences between respondents who had undergone at least one laparoscopic surgery (n* *=* *210) compared to those without a surgical history (n* *=* *30) for the sexual health outcomes. The 30 respondents without a surgical history were removed from the analyses to avoid potential confounding influence. GPower priori power analysis advised that 208 responses are required for linear regression models to achieve a power of 0.95, *P*≤0.05 (Faul *et al.*, 2007; Mayr *et al.*, 2007), indicating that the remaining sample of 210 responses provides sufficient power. Spearman’s Rho bivariate correlations were conducted to assess the associations between the outcome measures and endometriosis-specific variables. Hierarchical multiple regression analysis (MRA) was used to examine the unique contribution of endometriosis-related symptoms (step one), treatment (step two), and HRQoL (step three) have in influencing sexual function in individuals living with endometriosis. Individual regression models were conducted to determine the relative contribution of the examined factors in influencing the different components of sexual function and to extend the findings past that of sexual pain alone. The conceptual model (order of entry) was based on a review of the literature which indicates that endometriosis-related symptoms (block one: e.g. endometriosis-related pain, dysmenorrhea, dyspareunia) and treatment (block two: e.g. number of surgeries, hormonal treatment) have been found to influence HRQoL (block three). Hormonal therapy was included as a treatment of interest as preliminary analyses noted significant differences between participants receiving and not receiving hormonal treatment for level of endometriosis-related pain and HRQoL. HRQoL was entered in the final step (block three) to determine whether HRQoL independently influences sexual function in individuals with endometriosis. Multicollinearity analyses indicated that both the variance inflation factor (VIF) and tolerance scores (TS) were within acceptable ranges: VIF ≤ 3 and TS ≥ 1. Statistical significance was assumed at *P* ≤ 0.05.

### Ethics approval

Ethics approval was provided by the University of Tasmania Human Research Ethics Committee (reference: H0026906) for national and international respondents. Participants were provided with full study details prior to starting the survey and informed consent was indicated by submission of completed surveys.

## Results

The average endometriosis-related symptom duration reported by the sample was 14.8 years (SD* *=* *7.79), with 49.5% of the sample indicating a disease stage of moderate (Stage III) to severe (Stage IV). The average number of laparoscopic procedures was 2 (SD* *=* *1.48), ranging from one to nine surgeries. The average duration following the most recent laparoscopic surgery was 24.7 months prior to survey completion (SD* *=* *32.94), ranging from 1 month to 200 months. Fifty-five percent of the respondents (n* *=* *132) self-reported using hormonal therapy as a treatment for endometriosis (e.g. progestogens, GnRH analogues, combined contraceptives). Participants experienced an average of 8.9 symptoms (SD* *=* *3.10), ranging from one to 16 symptoms. The average level of endometriosis-related pain was 5.41 (SD* *=* *2.51), ranging from 1 to 10 where 0 equals no pain and 10 equals worst pain imaginable. Endometriosis-related pain was statistically significantly positively correlated with the length of time following the most recent laparoscopic surgery, with small effect size, indicating that higher pain levels are linked to longer gaps between surgeries. Level of endometriosis-related pain did not differ significantly for participants receiving hormonal therapy (Mean =* *5.41, SD* *=* *2.17) and those not receiving hormonal therapy (Mean =* *5.42, SD* *=* *2.83), *t*(208)=0.04, *P *=* *0.970. Further participant characteristics are outlined in Table 1.

**Table 1.** Participants demographic and endometriosis-related characteristics.

	Frequency	Percent (%)
Country of residence		
Australia	184	87.6
Oceania (e.g. New Zealand, Papua New Guinea)	5	2.4
UK	9	4.3
North America	12	5.7
Highest level of academic attainment		
High school or below	30	14.3
Vocational certificate/qualification	50	23.8
Bachelor degree	78	37.1
Postgraduate degree	52	24.8
Employment status		
Student	35	16.7
Employed, part-time	36	17.6
Employed, full-time	111	52.9
Unable to work due to health	27	12.8
Relationship status		
Single	58	27.6
Committed	87	41.4
Married, living together	65	31.0
Sexuality		
Gay, lesbian, or same gender attracted	21	10.0
Straight/heterosexual	161	76.7
Bisexual	17	8.1
Pansexual	7	3.3
Asexual	4	1.9
Perimenopause/menopause status		
I do not experience symptoms of perimenopause	164	78.0
I experience symptoms of perimenopause	21	10.0
I experienced a medical or surgical menopause	25	12.0
Reproductive history		
I have never tried to conceive	121	57.6
I have tried to conceive but have never been pregnant	17	8.1
I am currently trying to conceive	15	7.1
I am currently pregnant	2	1.0
I have been pregnant/no miscarriages	20	9.5
I have been pregnant/1 or more births/1 or more miscarriages	21	10.0
I have been pregnant/Nil live births	14	6.7

n* *=* *210.

### HRQoL outcomes

In the current endometriosis sample, six participants (2.9%) reported a full optimal health state of 11 111 and four participants reported an extremely poor health state of 44 444 (1.9%). As can be seen in Table 2, most participants experienced slight to moderate levels of daily pain and discomfort (72.4%), anxiety and/or depression (62.4%), difficulties engaging in their usual activities (64.8%), but no difficulties completing tasks of self-care such as washing and dressing themselves (69.0%). In terms of overall HRQoL, the EQ-5D-5L VAS score was used to represent the participant’s perception of their health at the time of survey completion where 0 equals worst health and 100 equals best health. In general, participants reported an average level of overall HRQoL (Mean =* *54.1, SD* *=* *23.27, range = 0.0–89.0). To allow a more meaningful comparison of the HRQoL reported by the current endometriosis sample compared to the general population, the health state scores were converted to population-based utility scores. The mean utility score was 0.76 (SD* *=* *0.22, range = 0.03–1.0) which is lower than the mean utility score reported in an Australian general population sample (Mean =* *0.9, SD* *=* *0.14; Norman *et al.*, 2023), reflecting poorer HRQoL in the current endometriosis sample than that reported in general population samples.

**Table 2.** Dimensions of health-related quality of life (EQ-5D-5L) in an endometriosis sample.

	Mobility, n (%)	Self-care, n (%)	Usual activities, n (%)	Pain/discomfort, n (%)	Anxiety/depression, n (%)
No problems	96 (45.7)	145 (69.0)	47 (22.4)	24 (11.4)	41 (19.5)
Slight problems	66 (31.4)	43 (20.5)	89 (42.4)	79 (37.6)	76 (36.2)
Moderate problems	38 (18.1)	22 (10.5)	47 (22.4)	73 (34.8)	55 (26.2)
Severe problems	10 (4.8)	0 (0.0)	16 (7.6)	25 (11.9)	20 (9.5)
Extreme problems/unable to do	0 (0.0)	0 (0.0)	11 (5.2)	9 (4.3)	18 (8.6)

n* *=* *210. EQ-5D-5L=EuroQol-5 Dimension.

Bivariate correlations were used to determine the relationships between endometriosis-related pain, laparoscopic surgery, and HRQoL (see Table 3). Statistically significant negative correlations were identified between the average level of endometriosis-specific pain experienced in the previous 7 days and the individual health dimensions, Health VAS, and utility score, with small to large effect sizes. This suggests that higher levels of endometriosis-related pain are significantly associated with lower levels of HRQoL as measured by the EQ-5D-5L (see Table 3).

**Table 3.** Standardized correlation matrix for associations between laparoscopic surgery, health-related quality of life (HRQoL), and sexual function.

Symptom	1.	2.	3.	4.	5.	6.	7.	8.	9.	10.	11.	12.	13.	14.	15.	16.	17.
1. PainVAS	–	0.059	0.044	0.001	0.001	0.001	0.001	0.001	0.001	0.001	0.830	0.203	0.132	0.012	0.025	0.001	0.017
2. SurgeryN	0.13	–	0.753	0.006	0.647	0.013	0.022	0.001	0.058	0.002	0.758	0.556	0.127	0.074	0.085	0.306	0.154
3. SurgeryD	**0.14**	0.02	–	0.046	0.001	0.001	0.016	0.036	0.013	0.001	0.416	0.823	0.954	0.419	0.937	0.499	0.947
4. Mobility	**−0.44**	**−0.19**	**0.14**	–	0.001	0.001	0.001	0.001	0.001	0.001	0.048	0.001	0.001	0.001	0.001	0.001	0.001
5. Self-Care	**−0.37**	−0.03	**0.23**	**0.58**	–	0.001	0.001	0.001	0.001	0.001	0.191	0.011	0.019	0.001	0.271	0.002	0.004
6. Activity	**−0.47**	**−0.17**	**0.30**	**0.59**	**0.50**	–	0.001	0.001	0.001	0.001	0.466	0.026	0.043	0.001	0.006	0.001	0.005
7. Pain/Disc	**−0.60**	**−0.16**	**0.17**	**0.62**	**0.49**	**0.57**	–	0.001	0.001	0.001	0.270	0.001	0.001	0.001	0.001	0.001	0.001
8. Anx/Dep	**−0.30**	**−0.23**	**0.15**	**0.33**	**0.32**	**0.34**	**0.41**	–	0.001	0.001	0.822	0.221	0.196	0.004	0.009	0.001	0.012
9. HealthVAS	**−0.48**	−0.13	**0.18**	**0.44**	**0.34**	**0.55**	**0.63**	**0.51**	–	0.001	0.024	0.001	0.001	0.001	0.001	0.001	0.001
10. Utility	**−0.59**	**−0.21**	**0.25**	**0.72**	**0.61**	**0.73**	**0.83**	**0.67**	**0.69**	–	0.155	0.001	0.001	0.001	0.001	0.001	0.001
11. Desire	−0.02	0.01	−0.06	**0.14**	0.09	0.05	0.08	0.02	**0.16**	0.10	–	0.001	0.001	0.001	0.001	0.001	0.001
12. Arousal	−0.09	−0.04	0.02	**0.23**	**0.17**	**0.15**	**0.26**	0.09	**0.24**	**0.27**	**0.68**	–	0.001	0.001	0.001	0.001	0.001
13. Lubrication	−0.10	−0.11	−0.01	**0.23**	**0.16**	**0.14**	**0.26**	0.09	**0.24**	**0.26**	**0.54**	**0.88**	–	0.001	0.001	0.001	0.001
14. Orgasm	**−0.17**	−0.12	0.06	**0.33**	**0.25**	**0.23**	**0.35**	**0.20**	**0.31**	**0.39**	**0.48**	**0.84**	**0.77**	–	0.001	0.001	0.001
15. Satisfact	**−0.16**	−0.12	0.01	**0.25**	0.08	**0.19**	**0.29**	**0.18**	**0.36**	**0.30**	**0.51**	**0.72**	**0.68**	**0.62**	–	0.001	0.001
16. SexPain	**−0.24**	−0.07	0.05	**0.34**	**0.22**	**0.26**	**0.38**	**0.23**	**0.38**	**0.43**	**0.41**	**0.66**	**0.66**	**0.64**	**0.60**	–	0.001
17. OverallSF	**−0.16**	−0.10	0.01	**0.31**	**0.20**	**0.19**	**0.33**	**0.17**	**0.34**	**0.35**	**0.71**	**0.94**	**0.90**	**0.87**	**0.82**	**0.79**	–

PainVAS  =  Average level of endometriosis-related pain experienced in the last 7 days; SurgeryN = Total number of laparoscopic surgeries; SurgeryD = Approximate length of time since most recent laparoscopic surgery (months); Mobility = EuroQol-5 Dimension Mobility domain; Self-care = EuroQol-5 Dimension Self-care domain; Activity = EuroQol-5 Dimension Usual Activities domain; Pain/Disc = EuroQol-5 Dimension Pain and Discomfort domain; Anx/Dep = EuroQol-5 Dimension Anxiety/Depression domain; HealthVAS = Self-rated HRQoL 0 is worst health imaginable and 100 is best health imaginable; Utility = EuroQol-5 Dimension Utility Norm score; Desire = Female Sexual Function Index desire subscale; Arousal = Female Sexual Function Index sexual arousal subscale; Lubrication = Female Sexual Function Index sexual lubrication subscale; Satisfaction. = Female Sexual Function Index sexual satisfaction subscale; SexPain = Female Sexual Function Index sexual pain subscale; OverallSF = Female Sexual Function Index overall level of sexual function; Significance level (2-tailed) reported in upper half of matrix in italics; Spearman’s Rho correlation presented in bottom half of matrix. Significant correlations in bold font (*P* < 0.05 to *P* < 0.001).

The number of laparoscopic surgeries was found to be statistically significantly negatively correlated with the mobility, usual activities, pain and discomfort, anxiety/depression domains and the utility score, with small effect sizes, with higher numbers of surgical procedures associated with poorer HRQoL in these areas. The length of time since the most recent laparoscopic surgery was found to be statistically significantly positively correlated with all aspects of the EQ-5D-5L, with small effect sizes, indicating that longer duration following surgery was associated with greater HRQoL in the current sample (see Table 3). Independent samples *t*-tests failed to indicate significant differences in HRQoL for those receiving hormonal therapy compared to those not undertaking hormonal therapy.

### Sexual function outcomes

As seen in Table 4, the average FSFI overall sexual function score was 16.89 (SD* *=* *9.65, range = 2.0–35.6), which falls within the clinical cut-off range of ≤26 and is suggestive of clinically significant female sexual dysfunction. The lowest level of function was noted for the pain subscale (Mean =* *2.24, SD* *=* *1.86, range = 0.0–6.0) and the highest level of function for the satisfaction subscale (Mean =* *3.25, SD* *=* *1.58, range = 0.80–6.0).

**Table 4.** Means and standard deviations for Female Sexual Function Index (FSFI) in women with endometriosis.

FSFI domain	*M* (SD)	Range
Desire	2.75 (1.35)	1.20–6
Arousal	2.78 (2.06)	0–6
Lubrication	3.18 (2.32)	0–6
Orgasm	2.69 (2.12)	0–6
Satisfaction	3.25 (1.58)	0.80–6
Pain	2.24 (1.86)	0–6
Overall sexual function	16.89 (9.65)	2–35.60

n* *=* 210.* M=Mean. Respondents are asked to rate their responses on a scale from 0 (No Sexual Activity) to 5 (Almost Always/Very High) or 1 (Very Dissatisfied) to 5 (Very Satisfied), with higher scores reflecting greater levels of sexual functioning in the individual domain. Overall FSFI score, ranging from 2 (dissatisfactory sexual function) to 36 (satisfactory sexual function).

Bivariate correlations were used to determine the relationships between endometriosis-related pain, laparoscopic surgery, and sexual function (see Table 3). The average level of endometriosis-related pain was statistically significantly negatively correlated with orgasmic function, sexual satisfaction, sexual pain, and overall level of sexual function, with small effect sizes. This suggests that higher levels of endometriosis-specific pain are linked to poorer orgasmic function, sexual satisfaction, overall sexual function, and greater sexual pain in the current sample (see Table 3). The number of laparoscopic surgeries and duration following the most recent laparoscopic surgery were not found to be statistically significantly correlated with sexual function in the current endometriosis sample (see Table 3).

Independent *t*-tests revealed several significant differences between participants receiving or not receiving hormonal therapy and all sexual function domains, with moderate effect sizes (excluding orgasmic function). Individuals undertaking hormonal therapy reported significantly poorer overall sexual function and poorer function in the domains of desire, arousal, lubrication, and sexual satisfaction compared to individuals not receiving hormonal therapy.

### Associations between HRQoL and sexual function outcomes

Bivariate correlations were used to determine the relationships between HRQoL (Health VAS, utility score) and sexual function (FSFI subscales, overall sexual function) (see Table 3). Statistically positive correlations were found between the Health VAS, utility score, individual FSFI subscales, and overall level of sexual function, with small to medium effect sizes, indicating that higher self-rated HRQoL and utility scores are associated with higher levels of sexual functioning.

### Correlates of sexual function for individuals with endometriosis

Hierarchical MRA was used to examine the unique contribution of endometriosis-specific symptoms associated with sexual functioning in existing literature (i.e. endometriosis-related pain, dysmenorrhea, dyspareunia, pain after sexual intercourse, intermenstrual bleeding, fertility concerns, fatigue, bloating, nausea, vulva pain, clitoral pain), treatment factors (i.e. number of laparoscopic surgeries, duration since most recent surgery in months, hormonal therapy), and HRQoL (Health VAS, utility score) on the specific domains of sexual functioning (i.e. sexual desire, sexual arousal, sexual lubrication, orgasmic function, sexual satisfaction, sexual pain) (please see Supplementary Data File S1 for hierarchical model equations).

As seen in Tables 5 and 6, the EQ-5D-5L Health VAS was a significant correlate in the final model for all the domains of sexual function, indicating that higher self-reported HRQoL is linked with greater function in the domains of desire, arousal, lubrication, orgasmic function, sexual satisfaction, and lower levels of sexual pain. As can be seen in Table 5, dysmenorrhea and intermenstrual bleeding were significant negative correlates of desire, arousal, and lubrication, with the presence of intermenstrual bleeding and dysmenorrhea associated with poorer function in these domains. Dysmenorrhea and dyspareunia were also indicated as a significant negative correlate in the final model for sexual satisfaction indicating that the presence of dysmenorrhea is associated with significantly lower levels of orgasmic function and sexual satisfaction. Dyspareunia and hormonal therapy were identified as significant negative correlates of arousal and lubrication, with the presence of dyspareunia and undertaking hormonal therapy associated with poorer function in the domains of sexual arousal and lubrication (see Table 5). Hormonal therapy was also indicated as a significant negative correlate of sexual satisfaction and sexual pain, with the use of hormonal therapy associated with significantly lower levels of satisfaction and greater sexual pain (see Table 6). Intermenstrual bleeding and nausea were found to be significant negative correlates of sexual pain, with the presence of intermenstrual bleeding and nausea associated with significantly higher levels of sexual pain (see Table 6). The length of time since the most recent laparoscopic surgery was identified as a significant negative correlate of sexual desire, with greater duration post-surgery associated with poorer function in the domain of sexual desire (see Table 5).

**Table 5.** Hierarchical multiple regression analyses for correlates of FSFI subscales of sexual desire, sexual arousal, and sexual lubrication in individuals with endometriosis.

Correlate	Sexual desire				Sexual arousal				Sexual lubrication			
	*b*	*β*	*t*	*P*	*b*	*β*	*t*	*P*	*b*	*β*	*t*	*P*
Step 1^a^												
Pain VAS	−0.01	−0.08	−1.06	0.290	−0.01	−0.11	−1.49	0.137	−0.01	−0.13	−1.74	0.084
Dysmenorrhea	−0.59	−0.22	−2.99	**0.003**	−0.97	−0.24	−3.41	**0.001**	−0.97	−0.21	−2.97	**0.003**
Dyspareunia	−0.53	−0.19	−2.17	**0.031**	−0.96	−0.24	−2.77	**0.006**	−0.72	−0.16	−1.79	0.074
Pain after sex	−0.25	−0.09	−1.05	0.297	0.20	0.05	0.56	0.572	0.32	0.07	0.80	0.425
IM bleeding	−0.64	−0.20	−2.76	**0.006**	−0.87	−0.18	−2.62	**0.010**	−0.70	−0.13	−1.81	0.072
Fertility concerns	−0.04	−0.01	−0.13	0.896	0.11	0.02	0.25	0.797	0.63	0.09	1.30	0.197
Fatigue	−0.27	−0.07	−0.90	0.372	−0.49	−0.09	−1.15	0.252	−0.44	−0.07	−0.89	0.374
Bloating	0.40	0.11	1.46	0.147	−0.13	−0.02	−0.34	0.738	−0.49	−0.08	−1.07	0.284
Nausea	0.05	0.02	0.23	0.818	−0.46	−0.11	−1.45	0.148	−0.35	−0.07	−0.96	0.339
Vulva pain	0.18	0.06	0.77	0.445	0.11	0.03	0.33	0.739	0.03	0.01	0.09	0.925
Clitoral pain	0.43	0.10	1.21	0.229	0.33	0.05	0.65	0.514	0.37	0.05	0.63	0.529
Step 2^b^												
Pain VAS	−0.01	−0.10	−1.26	0.210	−0.01	−0.11	−1.53	0.127	−0.01	−0.13	−1.73	0.085
Dysmenorrhea	−0.54	−0.20	−2.69	**0.008**	−0.85	−0.21	−2.97	**0.003**	−0.83	−0.18	−2.50	**0.013**
Dyspareunia	−0.49	−0.18	−1.99	**0.048**	−0.83	−0.20	−2.39	**0.018**	−0.56	−0.12	−1.38	0.170
Pain after sex	−0.26	−0.09	−1.02	0.309	0.33	0.08	0.92	0.357	0.50	0.11	1.22	0.224
IM bleeding	−0.60	−0.19	−2.48	**0.014**	−0.82	−0.17	−2.38	**0.019**	−0.67	−0.13	−1.67	0.097
Fertility concerns	−0.09	−0.02	−0.31	0.757	−0.04	−0.01	−0.10	0.917	0.43	0.06	0.88	0.379
Fatigue	−0.28	−0.07	−0.92	0.357	−0.52	−0.09	−1.23	0.222	−0.47	−0.07	−0.96	0.337
Bloating	0.39	0.10	1.40	0.163	−0.07	−0.01	−0.18	0.856	−0.41	−0.06	−0.89	0.375
Nausea	−0.15	−0.05	0.66	0.509	−0.36	−0.08	−1.11	0.267	−0.26	−0.05	−0.69	0.491
Vulva pain	0.19	0.07	0.82	0.415	0.12	0.03	0.35	0.728	0.04	0.01	0.11	0.911
Clitoral pain	0.34	0.08	0.94	0.347	0.21	0.03	0.41	0.680	0.26	0.03	0.43	0.666
Number of LES	0.06	0.05	0.66	0.509	−0.05	−0.03	−0.40	0.689	−0.11	−0.06	−0.77	0.444
Time since last LES	−0.01	−0.12	−1.73	0.085	−0.01	−0.06	−0.92	0.359	−0.01	−0.06	−0.92	0.357
Hormonal therapy	−0.34	−0.12	−1.64	0.101	−0.71	−0.17	−2.42	**0.017**	−0.87	−0.19	−2.56	**0.011**
Step 3^c^												
Pain VAS	−0.01	−0.02	−0.20	0.841	0.01	0.02	0.18	0.856	−0.01	−0.01	−0.04	0.964
Dysmenorrhea	−0.55	−0.20	−2.81	**0.005**	−0.91	−0.22	−3.26	**0.001**	−0.90	−0.19	−2.78	**0.006**
Dyspareunia	−0.47	−0.17	−1.93	0.055	−0.74	−0.18	−2.17	**0.031**	−0.45	−0.10	−1.13	0.261
Pain after sex	−0.32	−0.12	−1.30	0.194	0.23	0.05	0.64	0.520	0.39	0.08	0.97	0.335
IM bleeding	−0.64	−0.20	−2.68	**0.008**	−0.93	−0.20	−2.74	**0.007**	−0.78	−0.15	−2.00	**0.047**
Fertility concerns	−0.05	−0.01	−0.17	0.865	0.01	0.01	0.01	0.990	0.48	0.07	1.01	0.315
Fatigue	−0.24	−0.06	−0.82	0.413	−0.44	−0.07	−1.06	0.289	−0.38	−0.06	−0.80	0.426
Bloating	0.45	0.12	1.63	0.104	−0.04	−0.01	−0.11	0.907	−0.39	−0.06	−0.86	0.389
Nausea	−0.20	−0.07	−0.91	0.362	−0.26	−0.06	−0.81	0.416	−0.15	−0.03	−0.40	0.692
Vulva pain	0.21	0.07	0.92	0.360	0.16	0.04	0.49	0.625	0.09	0.02	0.24	0.810
Clitoral pain	0.42	0.10	1.18	0.239	0.44	0.07	0.86	0.388	0.52	0.07	0.87	0.384
Number of LES	0.08	0.06	0.83	0.405	0.01	0.01	0.06	0.950	−0.05	−0.02	−0.30	0.760
Time since last LES	−0.01	−0.15	−2.09	**0.038**	−0.01	−0.11	−1.64	0.102	−0.01	−0.11	−1.62	0.106
Hormonal therapy	−0.36	−0.13	−1.77	0.078	−0.73	−0.18	−2.52	**0.012**	−0.88	−0.19	−2.65	**0.009**
Health VAS	0.02	0.25	2.65	**0.009**	0.02	0.21	2.37	**0.019**	0.02	0.19	2.12	**0.035**
Utility score	−0.29	−0.05	−0.47	0.637	0.83	0.09	0.96	0.335	1.08	0.11	1.08	0.282

*^a^* =Endometriosis-related symptoms *^b^*=Endometriosis-related symptoms, Treatment *^c^*=Endometriosis-related symptoms, Treatment, Health-relation Quality of Life FSFI=Female Sexual Function Index Pain VAS=Pain Visual Analogue Scale (Average level of endometriosis-specific pain experienced in the last 7-day period); IM Bleeding=Intermenstrual Bleeding; Number of LES=Total number of laparoscopic surgeries; Time Since last LES=Approximate duration since last laparoscopic surgery (months); Health VAS=Self-Rated Health-Related Quality of Life (HRQoL) where 0 is worst health imaginable and 100 is best health imaginable; Utility Score=EuroQol-5 Dimension (EQ-5D-5L) populated normed score; *P*=0.05 to 0.001; Significant correlates noted in bold font.

**Table 6.** Hierarchical multiple regression analyses for correlates of FSFI subscales of orgasmic function, sexual satisfaction, and sexual pain in individuals with endometriosis.

Correlate	Orgasmic function				Sexual satisfaction				Sexual pain			
	*b*	*β*	*t*	*P*	*b*	*β*	*t*	*P*	*b*	*β*	*t*	*P*
Step 1^a^												
Pain VAS	−0.02	−0.23	−3.12	**0.002**	−0.02	−0.27	−3.60	**0.001**	−0.01	−0.17	−2.27	**0.024**
Dysmenorrhea	−0.95	−0.23	−3.23	**0.001**	−0.67	−0.21	−3.02	**0.003**	−0.30	−0.08	−1.19	0.237
Dyspareunia	−0.98	−0.23	−2.71	**0.007**	−0.79	−0.25	−2.93	**0.004**	−0.50	−0.13	−1.57	0.117
Pain after sex	0.11	0.03	0.30	0.765	0.15	0.05	0.55	0.581	0.02	0.01	0.07	0.948
IM bleeding	−0.73	−0.15	−2.10	**0.037**	−0.22	−0.06	−0.85	0.395	−0.75	−0.17	−2.47	**0.014**
Fertility concerns	0.46	0.07	1.05	0.295	0.40	0.09	1.23	0.220	0.12	0.02	0.31	0.757
Fatigue	0.70	0.12	1.58	0.116	−0.31	−0.07	−0.92	0.360	−0.29	−0.06	−0.76	0.450
Bloating	0.12	0.02	0.30	0.761	0.25	0.06	0.82	0.411	0.13	0.03	0.36	0.719
Nausea	−0.52	−0.12	−1.58	0.116	0.08	0.02	0.31	0.754	−0.65	−0.17	−2.28	**0.024**
Vulva pain	−0.31	−0.07	−0.88	0.382	−0.10	−0.03	−0.36	0.716	−0.62	−0.15	−2.02	**0.045**
Clitoral pain	0.23	0.03	0.42	0.671	0.20	0.04	0.50	0.616	−0.25	−0.04	−0.54	0.590
Step 2^b^												
Pain VAS	−0.02	−0.22	−2.86	**0.005**	−0.02	−0.27	−3.57	**0.001**	−0.01	−0.17	−2.30	**0.022**
Dysmenorrhea	−0.91	−0.22	−3.03	**0.003**	−0.56	−0.18	−2.53	**0.012**	−0.22	−0.06	−0.85	0.395
Dyspareunia	−0.89	−0.21	−2.42	**0.017**	−0.67	−−0.21	−2.47	**0.014**	−0.41	−0.11	−1.29	0.199
Pain after sex	0.24	0.06	0.62	0.534	0.30	0.09	1.07	0.285	0.10	0.03	0.30	0.763
IM bleeding	−0.87	−0.18	−2.38	**0.018**	−0.21	−0.06	−0.78	0.437	−0.72	−0.17	−2.29	**0.023**
Fertility concerns	0.34	0.05	0.76	0.449	0.25	0.05	0.77	0.442	0.01	0.01	0.04	0.971
Fatigue	0.73	0.12	1.62	0.106	−0.33	−0.08	−0.99	0.319	−0.31	−0.06	−0.80	0.427
Bloating	0.19	0.03	0.45	0.651	0.33	0.08	1.06	0.291	0.16	0.03	0.45	0.657
Nausea	−0.61	−0.14	−1.80	0.074	−0.13	0.04	0.52	0.607	−0.58	−0.15	−1.97	**0.035**
Vulva pain	−0.30	−0.07	−0.85	0.399	−0.09	−0.03	−0.36	0.722	−0.61	−0.15	−1.99	**0.047**
Clitoral pain	0.34	0.05	0.63	0.533	0.13	0.02	0.33	0.745	−0.33	−0.05	−0.69	0.490
Number of LES	−0.26	−0.14	−1.89	0.061	−0.11	−0.08	−1.09	0.277	−0.03	−0.02	−0.26	0.794
Time since last LES	−0.01	−0.02	−0.22	0.826	−0.01	−0.05	−0.76	0.433	−0.01	−0.06	−0.95	0.343
Hormonal therapy	−0.26	−0.06	−0.82	0.412	−0.63	−0.20	−2.75	**0.007**	−0.49	−0.12	−1.81	0.071
Step 3^c^												
Pain VAS	−0.01	−0.08	−0.93	0.354	−0.01	−0.13	−1.63	0.104	−0.01	0.01	0.02	0.987
Dysmenorrhea	−0.98	−0.23	−3.35	**0.001**	−0.60	−0.19	−2.83	**0.005**	−0.30	−0.08	−1.20	0.231
Dyspareunia	−0.78	−0.19	−2.17	**0.031**	−0.62	−0.20	−2.38	**0.018**	−0.30	−0.08	−1.00	0.321
Pain after sex	0.12	0.03	0.33	0.737	0.19	0.06	0.70	0.488	−0.03	−0.01	−0.11	0.913
IM bleeding	−0.98	−0.20	−2.77	**0.006**	−0.29	−0.08	−1.13	0.261	−0.85	−0.20	−2.83	**0.005**
Fertility concerns	0.39	0.06	0.90	0.371	0.32	0.07	1.01	0.311	0.08	0.01	0.21	0.831
Fatigue	0.82	0.14	1.88	0.062	−0.26	−0.06	−0.84	0.403	−0.21	−0.04	−0.57	0.567
Bloating	0.21	0.04	0.51	0.614	0.41	0.10	1.38	0.168	0.20	0.04	0.59	0.558
Nausea	−0.50	−0.11	−1.51	0.134	−0.23	−0.07	0.94	0.348	−0.47	−0.15	−1.89	**0.043**
Vulva pain	−0.25	−0.06	−0.73	0.465	−0.06	−0.02	−0.22	0.825	−0.56	−0.14	−1.93	**0.040**
Clitoral pain	0.60	0.09	1.12	0.267	0.30	0.06	0.77	0.441	−0.05	−0.01	−0.12	0.909
Number of LES	−0.19	−0.10	−1.40	0.164	−0.07	−0.05	−0.75	0.457	0.04	0.02	0.36	0.721
Time since last LES	−0.01	−0.07	−0.99	0.319	−0.01	−0.10	−1.51	0.132	−0.01	−0.13	−1.97	0.050
Hormonal therapy	−0.27	−0.06	−0.88	0.379	−0.66	−0.21	−3.01	**0.003**	−0.51	−0.13	−1.99	**0.048**
Health VAS	0.10	0.21	2.33	**0.021**	0.02	0.34	3.93	**0.001**	0.02	0.30	3.46	**0.001**
Utility score	1.07	0.11	1.19	0.237	−0.11	−0.02	−0.17	0.87	0.90	0.11	1.18	0.241

*^a^* =Endometriosis-related symptoms *^b^*=Endometriosis-related symptoms, Treatment *^c^*=Endometriosis-related Symptoms, Treatment, Health-related Quality of Life FSFI=Female Sexual Function Index Pain VAS=Pain Visual Analogue Scale (Average level of endometriosis-specific pain experienced in the last 7-day period); IM Bleeding=Intermenstrual Bleeding; Number of LES=Total number of laparoscopic surgeries; Time Since last LES=Approximate duration since last laparoscopic surgery (months); Health VAS=Self-Rated Health-Related Quality of Life (HRQoL) where 0 is worst health imaginable and 100 is best health imaginable; Utility Score=EuroQol-5 Dimension (EQ-5D-5L) populated normed score; *P*=0.05 to 0.001; Significant correlates noted in bold font.

### Associations between dyspareunia and the FSFI sexual pain domain

Noting that the presence of dyspareunia was not found to be a significant correlate of the FSFI sexual pain domain, bivariate correlations were used to explore whether the distress associated with the experience of dyspareunia may be of greater relevance than its presence alone. A statistically negative correlation was found between the level of distress associated with the presence of dyspareunia and the sexual pain domain, with moderate effect size (*r *=* −*0.38, *P *=* *0.001), with higher levels of distress regarding the experience of dyspareunia linked to greater levels of sexual pain (NB: lower scores on FSFI sexual pain subscale equal greater sexual pain).

## Discussion

The current study aimed to investigate HRQoL and sexual function in individuals with symptomatic endometriosis and the influence of laparoscopic surgery on these outcomes. The hypothesis that HRQoL and sexual function would be significantly negatively associated with the length of time following laparoscopic surgery was only partially supported, with a significant negative association only identified for HRQoL. Partial support was found for the hypothesis that a higher number of laparoscopic surgeries would be significantly negatively correlated with HRQoL and sexual function, with a significant negative association found between HRQoL and number of surgeries only.

Of note, level of endometriosis-related pain and HRQoL were not found to be significantly influenced by hormonal therapies. However, sexual function was found to be negatively influenced by hormonal therapies. This finding may be associated with the surgical focus on the current endometriosis sample, whereby laparoscopic surgery was a key inclusion criterion. Previous research has found that surgery may be necessary for one in three individuals with endometriosis where hormonal therapies are ineffective (Vercellini *et al.*, 2012). Efficacy of hormonal therapies has also been linked to disease stage, with individuals with no history of surgical intervention and early stage endometriosis showing similarities in treatment responsiveness (Jensen *et al.*, 2018). Much of the current sample self-reported a disease stage of moderate to severe, potentially reflecting that both engagement in surgery and disease stage may account for the current findings that use of hormonal therapy was linked to lowered levels of sexual function. The surgical focus and disease stage of the current sample may also explain the nonsignificant differences in current levels of endometriosis-related pain noted between those using or not using hormonal therapies currently. Reductions in therapeutic effectiveness of hormonal therapies have been associated with limited efficacy in reducing endometriosis-related pain, intolerable side effects, increased intermenstrual bleeding, and lowered sexual desire (Berlanda *et al.*, 2017). The current finding that the use of hormonal therapy was a significant negative correlate of sexual arousal, lubrication, sexual pain, and satisfaction appears consistent with prior findings indicating that, while hormonal therapies are effective for many individuals with endometriosis, they are linked to decreased sexual function in some individuals.

The findings from the current study add further evidence that HRQoL is significantly lower in individuals with endometriosis compared to the Australian public, as measured by the EQ-5D-5L norms (Norman *et al.*, 2023). Poorer HRQoL was especially prevalent in individuals experiencing higher levels of endometriosis-related pain, with poorer HRQoL noted across all individual domains of the EQ-5D-5L, the Health VAS, and utility score. It is also important to note that the current sample had undergone at least one laparoscopic surgery, meaning that the above findings may only be representative of the association between pain and HRQoL of individuals who have received surgical treatment. As such, further research with a greater sample of individuals who have not undergone laparoscopic surgery is required to gain further understanding of the potential influence of surgery on HRQoL outcomes. Future research that measures fluctuations in HRQoL from pre-surgical intervention to regular follow-up intervals is also recommended.

The results indicated that a higher number of laparoscopic surgeries for the treatment of endometriosis was associated with significantly poorer HRQoL in the domains of mobility, usual activity, pain and discomfort, anxiety/depression, and overall HRQoL (utility score). Given the recurrent and pervasive nature of endometriosis, it may be expected that surgical symptom management may be offered more frequently to those with greater physical impairment or disease stage (Pessoa de Farias Rodrigues *et al.*, 2020; Martínez-Zamora *et al.*, 2021; Laguerre *et al.*, 2022). Certainly, within the current sample, over 50% of the sample who reported having at least one laparoscopic surgery self-reported a disease stage of moderate or greater. However, pre-existing research has failed to indicate a clear connection between endometriosis disease stage and HRQoL or surgical outcomes (Leonardi *et al.*, 2020a; Van Niekerk *et al.*, 2022a). Similarly, Warzecha *et al.* (2020) reported that the presence of common endometriosis-related symptoms (e.g. dysmenorrhea, dyspareunia), type or number of laparoscopic procedures, and psychological wellbeing were not significantly correlated with endometriosis disease stage. They noted that painful defecation and infertility were the only endometriosis-related symptoms positively associated with disease stage, concluding that endometriosis-related symptoms are poorly predictive of disease stage (Warzecha *et al.*, 2020).

Research into the sexual response cycle provides valuable insight into the likelihood that the interplay between psychological and physical factors may act to inhibit the sexual response cycle, negatively impacting the individuals’ enjoyment of and willingness to engage in sexual activity (Fairbanks *et al.*, 2017; Della Corte *et al.*, 2020; Vannuccini *et al.*, 2023). A relatively consistent pattern of endometriosis-related symptoms (i.e. dysmenorrhea, dyspareunia, intermenstrual bleeding) were identified as significant negative correlates across multiple domains of sexual function. Endometriosis-related pain and the presence of nausea were also identified as significant negative correlates in specific domains of sexual function.

Dysmenorrhea was a significant negative correlate of sexual function across all subscales of sexual function, except for sexual pain. The significant negative impact of dysmenorrhea on sexual functioning noted in the current study supports the findings of Armour *et al.*, (2020) and Stokes *et al.* (2023) where individuals with severe or regular dysmenorrhea reported difficulties in their sexually intimate relationships. The results of the current study, and those of Armour *et al.*, (2020) and Stokes *et al.* (2023), contradict the assumption that the presence of dysmenorrhea is less impactful on sexual function than symptoms such as dyspareunia as it is cyclical in nature and therefore would only be of concern during menstruation (Pluchino *et al.*, 2016).

The presence of dyspareunia was also found to be a significant negative correlate of sexual function in the domains of desire, arousal, orgasmic function, and satisfaction. This finding is consistent with previous research that has highlighted the negative association between dyspareunia and sexual function in individuals with endometriosis (van Poll *et al.*, 2020). The further evidence that dyspareunia is a negative correlate of sexual function, but not specifically in the domain of sexual pain, in individuals with endometriosis highlights the importance of a clinical assessment that provides an opportunity for the individual to discuss their unique symptom presentation and overall sexual response profile with treatment providers. It may be that the presence of dyspareunia inhibits the initial phase of the sexual response cycle (i.e. desire to be intimate), leading to reduced engagement in sexual intimacy. A sexual response screener that focuses solely on the presence of pain during penetrative intercourse may not adequately account for the true impact of dyspareunia on sexual function. Support for extending the assessment beyond the presence or absence of dyspareunia alone is supported by the current finding that greater distress associated with the experience of dyspareunia is significantly linked to greater dysfunction in the sexual pain domain of the FSFI. The experience of dyspareunia has been linked to the experience of negative self-perception, feelings of guilt and inadequacy, and relational distress (Fritzer *et al.*, 2013), further emphasizing the importance of understanding how distressing this symptom is for the person diagnosed. Certainly, Facchin *et al.*, (2021) note that although dyspareunia is a common symptom of endometriosis, the subjective experience of sexual pain remains overlooked in individuals with endometriosis, particularly during patient–doctor interactions. The researchers argue that dyspareunia has a more pervasive impact on sexual function than adequately accounted for and warrants greater clinical attention (Facchin *et al.*, 2021). This is supported by the findings of Schubert *et al.*, (2023) who reported significant improvements in dyspareunia, at a 2-year follow-up time point, in a prospective cohort of 34 women with persistent pelvic pain who had engaged in pelvic physiotherapy.

The unique impact of nausea on the experience of sexual pain is consistent with the finding of reduced sexual function in non-endometriosis samples (Millheiser, 2012) and also supports prior findings regarding the presence of nausea as an endometriosis-related symptom worthy of consideration (Hansen *et al.*, 2014; Evans *et al.*, 2021). Although menstrual irregularities and pelvic pain are often referenced as key symptoms of endometriosis, interestingly over half of the current sample indicated the presence of nausea and its presence was associated with a moderate amount of distress. The experience of nausea may also be related to the process of central sensitization that may occur for individuals with endometriosis, with Evans *et al.* (2021) finding that abdominal pain, allover body aches, fatigue, and nausea are predictive of functional pain disability in individuals with endometriosis. Hansen *et al.* (2014) reported that individuals with endometriosis frequently reported a constellation of seven symptoms that included painful urination and defecation, constipation or diarrhoea, fatigue, abdominal pain, irregular bleeding, and nausea which they termed ‘visceral syndrome’, which indicates that the findings in relation to nausea and sexual function warrant further attention.

The findings that specific endometriosis-related symptoms are correlates of sexual function domains highlights the role of multidisciplinary treatment approaches, such as sexual assessment and therapy, rather than relying on medical management alone as well as ensuring that treatment recommendations are person-centred, considering the individual’s unique endometriosis symptom profile (Evans *et al.*, 2019; Van Niekerk *et al.*, 2019, 2023). Adopting a holistic treatment approach would involve shifting the onus of care from that of medical professionals to a multidisciplinary team model (Leonardi *et al.*, 2020b; Armour *et al.*, 2022; O’Hara *et al.*, 2022). A multidisciplinary care approach would ensure that surgical intervention is followed by targeted treatment by other medical and allied health practitioners to focus on continued improvement of HRQoL and sexual function outcomes post-surgery. Engagement of multidisciplinary care teams may also be of benefit prior to surgery to assist in managing distress associated with surgical wait times or to provide additional coping and management strategies when further laparoscopic surgery is not deemed to be appropriate. The existing research into endometriosis treatment is largely in support of multidisciplinary management, particularly given the biological, psychological, and social implications of endometriosis, with the consensus being that changes are needed in the diagnosis and management of the condition for improvements in HRQoL to be realized (Della Corte *et al.*, 2020; Evans *et al.*, 2021).

There are some limitations of the current study that are worth noting. Firstly, the correlational research design means that causation cannot be inferred, and the findings from this study are instead useful in that they can help shape future longitudinal research. Sampling bias is also a limitation of the current study’s design, as the survey was advertised primarily through endometriosis-related social media platforms and gynaecology services. Attempts were made to mitigate the risk of sampling bias by engaging more than one social media platform, multiple gynaecology clinics, and opening the survey to international respondents. The survey advertisement was also shared by a variety of different health practitioners and networks (e.g. gynaecology, sonography, psychology, physiotherapy, nursing) to broaden participant reach. However, the use of an online survey allowed anonymous responses which aimed to increase the honesty of responses and reduce the risk of social desirability bias impacting the data. The endometriosis diagnosis and surgical history are self-reported only and given the survey design and anonymity of responses was not able to be medically verified. However, Shafrir *et al.* (2021) found that self-reported endometriosis diagnosis was ∼70% accurate and >90% accurate when asked if the diagnosis had been confirmed via laparoscopic surgery, as asked in the current study, concluding that self-reported data can be consider valid in endometriosis samples. The current sample primarily consisted of individuals in heteronormative relationships, meaning that the findings from this study are not generalizable to individuals in sexually diverse and/or non-heteronormative relationships. However, the use of questionnaires with largely gender-neutral and inclusive language facilitates participation from individuals in non-heteronormative relationships. The survey advertisements also used gender inclusive language to ensure that the experiences of gender diverse people with endometriosis could be accounted for. Future qualitative research focusing on HRQoL and sexual function and any associated changes following laparoscopic surgery is warranted to further understand the current preliminary findings. The current findings relate only to individuals with symptomatic endometriosis and may not be generalizable to individuals with endometriosis who have experienced full symptomatic relief following laparoscopic surgery. It is also noted that most of the sample reported a disease of Stage 3 or greater and therefore further research is required to determine whether the current findings may be influenced by disease stage.

The chosen measure of sexual function in individuals with endometriosis is an important consideration in clinical and research settings. A recent systematic review conducted by Oppenheimer *et al.* (2024) has found that while the FSFI can be used to discriminate between endometriosis subtypes and has efficacy in evaluating outcomes associated with medical treatment, it may lack specificity for measuring surgical outcomes and low scores may not necessarily be solely reflective of the impacts of endometriosis. In contrast, the use of the Sexual Activity Questionnaire (SAQ: Thirlaway *et al.*, 1996) was noted to have good responsiveness for measuring surgical outcomes in the domains of pleasure and discomfort, has been validated in an endometriosis sample, and allows for a measure of clinically important difference (Oppenheimer *et al.*, 2024). The use of the minimum clinically important difference allows for a closer focus on the domain of concern for the patient and can be used to guide symptom management (Thirlaway *et al.*, 1996). Consistent with the aim of the current study to explore overall sexual function rather than limiting the scope to the experience of dyspareunia, Oppenheimer *et al.* (2024) argue that the selected sexual function measure must account for the entire sexual response cycle and not be isolated to measurement of pain during penetration alone. While the FSFI does allow for an assessment of the full sexual response cycle, further development of endometriosis-specific scoring norms is recommended. In the absence of FSFI endometriosis-specific norms, use of the FSFI and SAQ together may provide a more comprehensive treatment measure for individuals with endometriosis. The findings from this study support the assertion that care for individuals with endometriosis must extend beyond attempting to alleviate the severity of pain alone and should instead represent a more holistic approach, particularly when considering sexual function and HRQoL. Early referral for assessment and management of sexual concerns is recommended as part of multidisciplinary endometriosis management. Multidisciplinary management of sexual function should address the individual domains of sexual function rather than a limited sexual pain focus. Future studies should look to build upon the small pool of existing research into the unique contribution of specific endometriosis-related symptom profiles on sexual function. Specifically, future research should look longitudinally at the impact of laparoscopic surgery on sexual function from a dyadic perspective, in both heteronormative and non-heteronormative relationships, with the aim of identifying key therapeutic areas for targeted intervention when working with individuals, or couples, with endometriosis.

## Supplementary Material

deae063_Supplementary_Data_File_S1

## Data Availability

Datasets are available on request: The raw data supporting the conclusions of this article will be made available by the authors on written request.
