# Supplementary material for: An evaluation of sexual function and health-related quality of life following laparoscopic surgery in individuals living with endometriosis
Source: Hum Reprod. 2024 Apr 1;39(5):992–1002. doi: 10.1093/humrep/deae063 (PMC11063542; doi:10.1093/humrep/deae063)
Supplement: deae063_Supplementary_Data_File_S1 [file deae063_supplementary_data_file_s1.pdf]

OXFORD
human reproduction
Human Reproduction, 2024, 1–2
https://doi.org/10.1093/humrep/deae063
Supplementary Data

Supplementary Data File S1

Hierarchical Multiple Regression Analyses Model Summary

Sexual Desire. On Step 1 of the hierarchical MRA (Multiple Regression Analysis), the absence/presence of individual endometriosis-related symptoms accounted for a significant 12% of the variance in sexual desire, $R^2 = 0.123$, $F(11, 190) = 2.42$, $P = 0.008$. On Step 2, treatment factors accounted for a nonsignificant 2.7% additional variance in sexual desire, $R^2 = 0.150$, $F(3, 187) = 1.97$, $P = 0.120$. On Step 3 of the model health-related quality of life (HRQoL) accounted for a significant 3.6% additional variance in sexual desire, $R^2 = 0.186$, $F(2, 185) = 4.12$, $P = 0.018$. In combination, the correlates accounted for 18.6% of the variance, $R^2 = 0.186$, adjusted $R^2 = 0.116$, $F(16, 185) = 2.65$, $P = 0.001$.

As can be seen in Table 5, when the absence/presence of certain endometriosis-related symptoms were entered on Step 1, dysmenorrhea, dyspareunia, and intermenstrual bleeding were significant correlates of sexual desire. When treatment factors were added at Step 2, dysmenorrhea, dyspareunia, and intermenstrual bleeding were significant correlates of sexual desire. The significant positive correlate of sexual desire in the final model was Health VAS (Visual Analogue Scale) with higher self-reported health associated with greater function in the domain of sexual desire. The significant negative predictors of sexual desire in the final model were dysmenorrhea, intermenstrual bleeding, and length of time since the most recent laparoscopic surgery, with the presence of intermenstrual bleeding and dysmenorrhea and greater duration post-surgery associated with poorer function in the domain of sexual desire.

Sexual Arousal. On Step 1 of the hierarchical MRA, the absence/presence of individual endometriosis-related symptoms accounted for a significant 19.8% of the variance in sexual arousal, $R^2 = 0.198$, $F(11, 190) = 4.25$, $P = 0.001$. On Step 2, treatment factors accounted for a nonsignificant additional 2.7% of the variance in sexual arousal, $R^2 = 0.225$, $F(3, 187) = 2.18$, $P = 0.092$. On Step 3 of the model HRQoL accounted for a significant additional 4.9% of the variance in sexual arousal, $R^2 = 0.274$, $F(2, 185) = 6.27$, $P = 0.002$. In combination, the correlates accounted for 27.4% of the variance, $R^2 = .274$, adjusted $R^2 = 0.211$, $F(16, 185) = 4.36$, $P = 0.001$.

As can be seen in Table 5, when the absence/presence of certain endometriosis-related symptoms were entered on Step 1, dysmenorrhea, dyspareunia, and intermenstrual bleeding were significant correlates of sexual arousal. When treatment factors were added at Step 2, dysmenorrhea, dyspareunia, intermenstrual bleeding, and undertaking hormonal therapy were significant correlates of sexual arousal. The significant positive correlate of sexual arousal in the final model was the Health VAS with higher self-reported health associated with greater function in the domain of sexual arousal. The significant negative correlates of sexual arousal in the final model were dysmenorrhea, dyspareunia, intermenstrual bleeding, and hormonal therapy with the presence of dysmenorrhea, dyspareunia, intermenstrual bleeding, and undertaking hormonal therapy associated with poorer function in the domain of sexual arousal.

Sexual Lubrication. On Step 1 of the hierarchical MRA, the absence/presence of individual endometriosis-related symptoms accounted for a significant 15.6% of the variance in sexual lubrication, $R^2 = 0.156$, $F(11, 190) = 3.18$, $P = 0.001$. On Step 2, treatment factors accounted for a nonsignificant 3.3% additional variance in sexual lubrication, $R^2 = 0.188$, $F(3, 187) = 2.51$, $P = 0.060$. On Step 3 of the model HRQoL accounted for a significant additional 4.7% of the variance in sexual lubrication, $R^2 = 0.235$, $F(2, 185) = 5.64$, $P = 0.004$. In combination, the correlates accounted for 23.5% of the variance, $R^2 = 0.235$, adjusted $R^2 = 0.169$, $F(16, 185) = 3.55$, $P = 0.001$.

As can be seen in Table 5, when the absence/presence of certain endometriosis-related symptoms were entered on Step 1, dysmenorrhea was the only significant correlate of sexual lubrication. When treatment factors were added at Step 2, dysmenorrhea, and hormonal therapy were significant correlates of sexual lubrication. The significant positive correlate of sexual lubrication in the final model was the Health VAS with higher self-reported health associated with greater function in the domain of sexual lubrication. The significant negative correlates of sexual lubrication in the final model were dysmenorrhea, intermenstrual bleeding, and hormonal therapy with the presence of dysmenorrhea, intermenstrual bleeding, and undertaking hormonal therapy associated with poorer function in the domain of sexual lubrication.

Orgasmic Function. On Step 1 of the hierarchical MRA, the absence/presence of individual endometriosis-related symptoms accounted for a significant 17.6% of the variance in orgasmic function, $R^2 = 0.176$, $F(11, 190) = 3.70$, $P = 0.001$. On Step 2, treatment factors accounted for a nonsignificant 1.8% additional variance in orgasmic function, $R^2 = 0.194$, $F(3, 187) = 1.36$, $P = 0.256$. On Step 3 of the model HRQoL accounted for a significant 5.5% additional variance in orgasmic function, $R^2 = 0.249$, $F(2, 185) = 6.81$, $P = 0.001$. In combination, the correlates accounted for 24.9% of the variance, $R^2 = .249$, adjusted $R^2 = 0.184$, $F(16, 184) = 3.84$, $P = 0.001$.

As can be seen in Table 6, when the absence/presence of endometriosis-related symptoms were entered on Step 1, endometriosis-related pain, dysmenorrhea, dyspareunia, and intermenstrual bleeding were the significant correlates of orgasmic function. When treatment factors were added at Step 2, endometriosis-related pain, dysmenorrhea, dyspareunia, and intermenstrual bleeding were significant correlates of orgasmic function. The significant positive correlate of orgasmic function in the final model was the Health VAS with higher self-reported health associated with greater function in the domain of orgasmic function. The significant negative correlates of orgasmic function in the final model were dysmenorrhea, dyspareunia, and intermenstrual bleeding, with the presence of dysmenorrhea, dyspareunia, and intermenstrual bleeding associated with poorer function in the domain of orgasmic function.

Sexual Satisfaction. On Step 1 of the hierarchical MRA, the absence/presence of individual endometriosis-related symptoms accounted for a significant 18.1% of the variance in sexual satisfaction, $R^2 = 0.181$, $F(11, 190) = 3.80$, $P = 0.001$. On Step 2, treatment factors accounted for a significant 3.7% additional variance in sexual satisfaction, $R^2 = 0.217$, $F(3, 187) = 2.93$, $P = 0.035$. On Step 3 of the model HRQoL accounted for a significant additional 7.8% of

the variance in sexual satisfaction, $R^2 = 0.296$, $F(2, 185) = 10.29$, $P = 0.001$. In combination, the correlates accounted for 29.6% of the variance, $R^2 = 0.296$, adjusted $R^2 = 0.235$, $F(16, 185) = 4.86$, $P = 0.001$.

As can be seen in Table 6, when the absence/presence of endometriosis-related symptoms were entered on Step 1, endometriosis-related pain, dysmenorrhea, and dyspareunia were the significant correlates of sexual satisfaction. When treatment factors were added at Step 2, endometriosis-related pain, dysmenorrhea, dyspareunia, and hormonal therapy were significant correlates of sexual satisfaction. The significant positive correlate of sexual satisfaction in the final model was the Health VAS with higher self-reported health associated with greater function in the domain of sexual satisfaction. The significant negative correlates of sexual satisfaction in the final model were dysmenorrhea, dyspareunia, and hormonal therapy with the presence of dysmenorrhea and dyspareunia and use of hormonal therapy associated with poorer function in the domain of sexual satisfaction.

Sexual Pain. On Step 1 of the hierarchical MRA, the absence/presence of individual endometriosis-related symptoms accounted for a significant 21.0% of the variance in sexual pain, $R^2 = 0.210$, $F(11, 190) = 4.58$, $P = 0.001$. On Step 2, treatment factors accounted for a nonsignificant 1.7% of the additional variance in sexual pain, $R^2 = 0.226$, $F(3, 187) = 1.35$, $P = 0.259$. On Step 3 of the model HRQoL accounted for a significant 9.1% additional variance in sexual pain, $R^2 = 0.317$, $F(2, 185) = 12.32$, $P = 0.001$. In combination, the correlates accounted for 31.7% of the variance, $R^2 = 0.317$, adjusted $R^2 = 0.258$, $F(16, 185) = 5.38$, $P = 0.001$.

As can be seen in Table 6, when the absence/presence of endometriosis-related symptoms were entered on Step 1, endometriosis-related pain, intermenstrual bleeding, nausea, and vulval pain were the significant correlates of sexual pain. When treatment factors were added at Step 2, endometriosis-related pain, intermenstrual bleeding, nausea, and vulval pain were significant correlates of sexual pain. The significant positive correlate of sexual pain in the final model was the Health VAS with higher self-reported health associated with lower levels of sexual pain. The significant negative correlates of sexual pain in the final model were intermenstrual bleeding, nausea, and hormonal treatment, with the presence of intermenstrual bleeding and nausea and use of hormonal therapy associated with greater sexual pain.
